# Putting a finger on histidine methylation

**DOI:** 10.1101/gad.351097.123

**Published:** 2023-08-01

**Authors:** Paul L. Boutz

**Affiliations:** Department of Biochemistry and Biophysics, Center for RNA Biology, Center for Biomedical Informatics, Wilmot Cancer Institute, University of Rochester School of Medicine and Dentistry, Rochester, New York 14620, USA

**Keywords:** methyltransferase, CARNMT1, methylation, histidine, mRNA, splicing, degradation, zinc finger protein, U2AF1, embryonic lethality, embryonic development

## Abstract

Specialized enzymes add methyl groups to the nitrogens of the amino acid histidine, altering the chemical properties of its imidazole ring and, in turn, the function of the modified (poly)peptide. In this issue of *Genes & Development*, Shimazu and colleagues (pp. 724–742) make the remarkable discovery that CARNMT1 acts as a dual-specificity histidine methyltransferase, modifying both the small-molecule dipeptide carnosine and a set of proteins, predominantly within RNA-binding C3H zinc finger (C3H ZF) motifs. As a result, CARNMT1 modulates the activity of its protein targets to affect RNA processing and metabolism, ultimately contributing an essential function during mammalian development.

## Abstract (precis)

In this Outlook, Boutz discusses a study in this issue of *Genes & Development* by Shimazu et al. that describes the role of CARMT1, a histidine methyltransferase, in RNA processing, commenting on CARMT1's substrate specificity and developmental essentiality.

Histidine methylation was discovered in 1967, but in comparison with other post-translational modifications such as arginine methylation, its biological functions have remained obscure until very recently. The two nitrogens in the imidazole ring of histidine (N1 and N3) are involved in hydrophobic interactions, confer affinity for divalent metal ions including zinc, and can switch from neutral to positively charged depending on their protonation state. Both can undergo methylation, which affects their biochemical properties (for review, see Jakobsson 2021).

The histidine methyltransferase enzymes in mammals have only recently been discovered. METTL9 methylates the N1 position of the HxH motif in the mammalian proteome (Davydova et al. 2021). Carnosine N-methyltransferase 1 (CARNMT1) methylates the dipeptide carnosine (Ala-His) also at the N1 position to produce anserine (Drozak et al. 2015). As its name would suggest, carnosine and its derivatives are highly abundant in skeletal muscle, where they function to maintain homeostasis by buffering pH, chelating metal ions, and acting as antioxidants (for review, see Boldyrev et al. 2013). In addition to carnosine, CARNMT1 can methylate multiple dipeptide and tripeptide sequences, suggesting that it may possess a broader target specificity (Drozak et al. 2015). In this issue of *Genes & Development*, Shimazu et al. (2023) began with a simple question: What other peptides does CARNMT1 methylate?

Testing the activity of recombinant CARNMT1 and METTL9 on short peptides corresponding to known N1 methylation sites within proteins, they found several histidines with up to 95% modification by CARNMT1. CARNMT1 and METTL9 were knocked out either individually or in combination. Each individual knockout (KO) reduced cellular N1-methylated histidine amounts by ∼50%, while the double knockout resulted in undetectable amounts of the modification, indicating that CARNMT1 and METTL9 likely share between them most of the histidine N1-methyltransferase activity in the cells tested.

In order to identify the protein substrates of CARNMT1, CARNMT1 knockout cells were incubated with or without recombinant CARNMT1 protein, and modified proteins were enriched and subjected to mass spectrometry. Using this methodology, 22 proteins were identified as CARNMT1 targets and several contained C3H zinc finger (C3H ZF) motifs, a type of RNA-binding domain that is found on many proteins involved in RNA metabolism and regulation (for review, see Hall 2005). About half of the identified methylation sites were located within C3H ZFs. These included the splicing factor U2AF1, which exhibited >99% methylation at H37 in the first of two C3H ZFs.

To gain insight into the physiological functions of C3H ZF methylation, Shimazu et al. (2023) turned to a well-characterized model of post-transcriptional regulation: TNF-α mRNA stability. In the 3′ UTR of mRNA encoding the proinflammatory TNF-α protein, binding of the C3H ZF proteins tristetraprolin (TTP) and Roquin promotes mRNA degradation (Carballo et al. 1998; Leppek et al. 2013). Methylation of both proteins in their C3H ZFs by CARNMT1 led to decreased coimmunoprecipitation of TNF-α mRNA. The half-life of TNF-α mRNA was almost doubled in the presence of CARNMT1. Further extending this observation, in a CARNMT1 KO macrophage cell line stimulated with lipopolysaccharide (LPS), TNF-α mRNA and protein levels were reduced in comparison with cells expressing CARNMT1. Together, the data suggest that CARNMT1 methylation of TTP and Roquin reduces the affinity of their C3H ZF domains for TNF-α mRNA, thereby stabilizing it and enhancing TNF-α protein expression during an inflammatory response.

Given the almost complete methylation of the first C3H ZF of the core splicing factor U2AF1 in the presence of CARNMT1, it is reasonable to assume that mRNA splicing patterns might be affected by U2AF1 methylation status. Indeed, almost 300 alternative splicing differences were detected by RNA-seq in CARNMT1 KO versus wild-type cells. Many of the affected events were cassette exons possessing weak 3′ splice sites with a preference for U versus C at the −3 position, reminiscent of exons differentially affected by U2AF1 S34F/Y mutations found in numerous cancers (Przychodzen et al. 2013; Ilagan et al. 2015). With additional experimental evidence, Shimazu et al. (2023) concluded that methylation of H37 in the first U2AF1 ZF by CARNMT1 enhances its affinity for weak 3′ splice sites. Thus, U2AF1 in the CARNMT1 KO cells behaves similarly to the U2AF1 S34F/Y mutants, raising a fascinating question: Do these cancer-associated mutations mimic a cell state in which CARNMT1 activity is physiologically down-regulated to execute a specific splicing program? It will be valuable to assess the methylation status of S34F/Y mutant U2AF1, as these mutations occur only three amino acids upstream of the methylated histidine. Is it possible that the mutations exert their effects by eliminating the substrate specificity for CARNMT1, or do they alter the conformation of the ZF to resemble the unmethylated motif? These questions will guide critical future experiments.

In contrast to METTL9, whose N1 methylation of its HxH consensus site alters the affinity for metal ions (Davydova et al. 2021), synthetic peptides representing a C3H ZF targeted by CARNMT1 showed no methylation-dependent difference in zinc ion affinity. However, there was evidence of a change in protein conformation affecting the binding mode. An outstanding question that will likely require structure-based approaches to address is how the methyl group alters the properties of C3H ZFs with regard to RNA binding at the molecular level. Are all C3H ZF methylation events functionally equivalent, or does the effect depend on other factors, such as motif context or the RNA substrate? The differences observed by Shimazu et al. (2023)—whose results indicate that TTP and Roquin binding to 3′ UTR sequences was diminished by CARNMT1 methylation, while methylation increased binding to weak 3′ splice sites by U2AF1—suggest the latter.

Intriguingly, carnosine but not anserine inhibited CARNMT1-dependent protein methylation, suggesting competition between substrates for limiting methyltransferase activity. Carnosine and anserine have been proposed to have exocrine functions when released from muscle during exercise (Boldyrev et al. 2013). CARNMT1 is the first known methyltransferase with dual substrate specificity, modifying both a small-molecule dipeptide and protein targets. This raises the intriguing possibility that a feedback loop linking exocrine activity of the dipeptide substrate, in competition with protein targets modulating extensive gene expression programs, centers on limiting CARNMT1 enzymatic activity. This opens a broad area for further research.

Finally, Shimazu et al. (2023) found that genetic ablation of CARNMT1 is embryonically lethal in mice. This was true also of homozygous catalytically dead mutant animals, definitively demonstrating a requirement for CARNMT1 methyltransferase activity for proper development. It was previously shown that synthesis of carnosine is dispensable for embryogenesis, indicating that it is the protein methylation targets that are essential (Wang-Eckhardt et al. 2020). In knockout embryos, alternative splicing changes similar to those seen in the knockout cell lines were observed. Although U2AF1 KO is also known to be embryonic-lethal, further analyses will be required to determine which pathways altered by lack of CARNMT1 activity are essential for development. Regardless of how its modifications are read out in the developing organism, the essentiality of CARNMT1 underscores the importance and scale of this novel area of biology that Shimazu et al. (2023) have uncovered.

## References

[GAD351097BOUC1] Boldyrev AA, Aldini G, Derave W. 2013. Physiology and pathophysiology of carnosine. Physiol Rev 93: 1803–1845. 10.1152/physrev.00039.2012 24137022

[GAD351097BOUC2] Carballo E, Lai WS, Blackshear PJ. 1998. Feedback inhibition of macrophage tumor necrosis factor-α production by tristetraprolin. Science 281: 1001–1005. 10.1126/science.281.5379.1001 9703499

[GAD351097BOUC3] Davydova E, Shimazu T, Schuhmacher MK, Jakobsson ME, Willemen H, Liu T, Moen A, Ho AYY, Małecki J, Schroer L, 2021. The methyltransferase METTL9 mediates pervasive 1-methylhistidine modification in mammalian proteomes. Nat Commun 12: 891. 10.1038/s41467-020-20670-7 33563959 PMC7873184

[GAD351097BOUC4] Drozak J, Piecuch M, Poleszak O, Kozlowski P, Chrobok L, Baelde HJ, de Heer E. 2015. UPF0586 protein C9orf41 homolog is anserine-producing methyltransferase. J Biol Chem 290: 17190–17205. 10.1074/jbc.M115.640037 26001783 PMC4498059

[GAD351097BOUC5] Hall TM. 2005. Multiple modes of RNA recognition by zinc finger proteins. Curr Opin Struct Biol 15: 367373. 10.1016/j.sbi.2005.04.004 15963892

[GAD351097BOUC6] Ilagan JO, Ramakrishnan A, Hayes B, Murphy ME, Zebari AS, Bradley P, Bradley RK. 2015. *U2AF1* mutations alter splice site recognition in hematological malignancies. Genome Res 25: 14–26. 10.1101/gr.181016.114 25267526 PMC4317169

[GAD351097BOUC7] Jakobsson ME. 2021. Enzymology and significance of protein histidine methylation. J Biol Chem 297: 101130. 10.1016/j.jbc.2021.101130 34461099 PMC8446795

[GAD351097BOUC8] Leppek K, Schott J, Reitter S, Poetz F, Hammond MC, Stoecklin G. 2013. Roquin promotes constitutive mRNA decay via a conserved class of stem-loop recognition motifs. Cell 153: 869–881. 10.1016/j.cell.2013.04.016 23663784

[GAD351097BOUC9] Przychodzen B, Jerez A, Guinta K, Sekeres MA, Padgett R, Maciejewski JP, Makishima H. 2013. Patterns of missplicing due to somatic U2AF1 mutations in myeloid neoplasms. Blood 122: 999–1006. 10.1182/blood-2013-01-480970 23775717 PMC3739042

[GAD351097BOUC10] Shimazu T, Yoshimoto R, Kotoshiba K, Suzuki T, Matoba S, Hirose M, Akakabe M, Sotohme Y, Sodeoka M, Ogura A, 2023. Histidine N1-position-specific methyltransferase targets C3H zinc finger proteins and modulates RNA metabolism. Genes Dev (this issue). 10.1101/gad.350755.123 PMC10546975 37612136

[GAD351097BOUC11] Wang-Eckhardt L, Bastian A, Bruegmann T, Sasse P, Eckhardt M. 2020. Carnosine synthase deficiency is compatible with normal skeletal muscle and olfactory function but causes reduced olfactory sensitivity in aging mice. J Biol Chem 295: 17100–17113. 10.1074/jbc.RA120.014188 33040025 PMC7863879

